# Evaluation of Plaque Imaging Techniques for Calcified Lesions Using MRI Termed Modified Liver Acquisition With Volume Acceleration (mLAVA)

**DOI:** 10.7759/cureus.104914

**Published:** 2026-03-09

**Authors:** Yoshiaki Kitazawa, Kiyofumi Yamada, Kisaki Amemiya, Yoshiaki Morita, Hiroharu Kataoka

**Affiliations:** 1 Department of Neurosurgery, National Cerebral and Cardiovascular Center, Suita, JPN; 2 Department of Pathology, National Cerebral and Cardiovascular Center, Suita, JPN; 3 Department of Radiology, National Cerebral and Cardiovascular Center, Suita, JPN

**Keywords:** calcified nodule, calcified plaque, carotid artery stenosis, mri, plaque imaging

## Abstract

As some calcified plaques are associated with an increased risk of future ischemic events, imaging evaluation of calcified lesions is clinically important. CT and CT angiography (CTA) are commonly used for the imaging evaluation of calcified lesions. However, the exposure to ionizing radiation has been associated with an increased lifetime risk of cancer, and the use of iodinated contrast media has the potential risk of renal dysfunction. In this study, we introduce the modified Liver Acquisition With Volume Acceleration (mLAVA) sequence, which is a novel MRI sequence for detecting carotid artery calcifications. We present two cases in which the mLAVA sequence identified high-risk calcified plaques and subsequently conducted carotid endarterectomy (CEA). The first case was a 54-year-old man with symptomatic carotid artery stenosis with a rim sign who underwent CEA. The second case was an 82-year-old man with asymptomatic carotid artery stenosis associated with a calcified nodule who also underwent CEA. In both cases, preoperative assessment of the calcified lesions was performed using the mLAVA sequence, and the imaging findings are presented. Our findings suggest that the mLAVA sequence may help preoperative evaluation of plaque imaging of calcified plaques as well as evaluation by CTA, particularly in patients with renal dysfunction or radiocontrast media allergy. Further advancements that enable precise visualization of fine calcified lesions would enhance their clinical utility.

## Introduction

Calcified plaques have been considered stable plaques with a low risk of stroke [1]. However, recent studies have demonstrated that certain calcified plaques, such as external membrane calcification (Rim sign) and calcified nodules (CNs), are associated with an increased risk of ischemic events [2-4]. Therefore, accurate detection and characterization of these high-risk calcified plaques are clinically important for preventing cerebral ischemic events and treatment planning in patients with carotid artery stenosis.

Evaluation of such calcified lesions has primarily relied on CT angiography (CTA). However, CTA has several limitations. Exposure to ionizing radiation has been associated with an increased lifetime risk of cancer, and the use of iodinated contrast media has a potential risk of contrast-induced nephropathy, which is particularly true for patients with carotid artery stenosis because they frequently have comorbidities such as renal dysfunction [5,6]. Therefore, a non-contrast and radiation-free imaging modality for detecting calcified carotid plaques would be clinically valuable.

MRI plaque imaging has been widely used to evaluate carotid plaque characteristics, especially unstable plaques. In addition, the modified Liver Acquisition With Volume Acceleration (mLAVA) sequence introduced in this study was useful for detecting calcified plaques and has the advantage of being acquired during the same imaging session with other MRI sequences. We report two cases of carotid artery stenosis with high-risk calcified plaques. Both cases were assessed using the mLAVA sequence before performing carotid endarterectomy (CEA), highlighting the importance of assessing calcified lesions.

## Case presentation

In this study, we present a novel MRI plaque imaging technique specifically designed for the assessment of calcification without contrast media, termed the mLAVA sequence. The original LAVA sequence is recognized as one of the fat-saturation T1-weighted imaging techniques using the three-dimensional fast gradient echo sequence for abdominal imaging [7]. We arranged a short TE (TE = 1.9 ms) and high-resolution LAVA sequence for carotid calcification imaging. Furthermore, image evaluation was performed using grayscale-inverted images to improve the detection of calcified lesions. The mLAVA sequence was performed using a SIGNA Premier 3.0-T scanner (GE Healthcare, Milwaukee, WI, USA). The following sequences were included in the protocol of this study: T1-weighted volume isotropic turbo spin echo acquisition (T1-CUBE) and three-dimensional time-of-flight magnetic resonance angiography (3D TOF-MRA) (Table 1).

**Table 1:** Sequence of mLAVA and T1-CUBE. mLAVA = modified Liver Acquisition With Volume Acceleration; T1-CUBE = T1-weighted volume isotropic turbo spin echo acquisition; TR = repetition time; TE = echo time; FA = flip angle; BW = bandwidth; FOV = field of view; NEX = number of excitations; ARC = autocalibrating reconstruction for Cartesian imaging

Parameter	mLAVA	T1 Cube
TR (msec)	4.4	678
TE (msec)	1.9	11
FA (deg)	5	Variable
BW (Hz/pixel)	520.8	434
FOV (mm)	200	240
Pixel size (mm)	0.6 × 0.8 × 1.0	0.8 × 0.9 × 1.6
Slice thickness (mm)	1	0.8
NEX	3	1
ARC	2	2
HyperSense		1.2
Number of slices	120	140
Scan time (s)	125	176

In this case report, we describe two patients with carotid artery stenosis who received preoperative MRI plaque imaging using the mLAVA sequence before CEA between April 2024 and September 2024 at the National Cerebral and Cardiovascular Center, Osaka, Japan.

Case 1: rim sign

**Figure 1:**
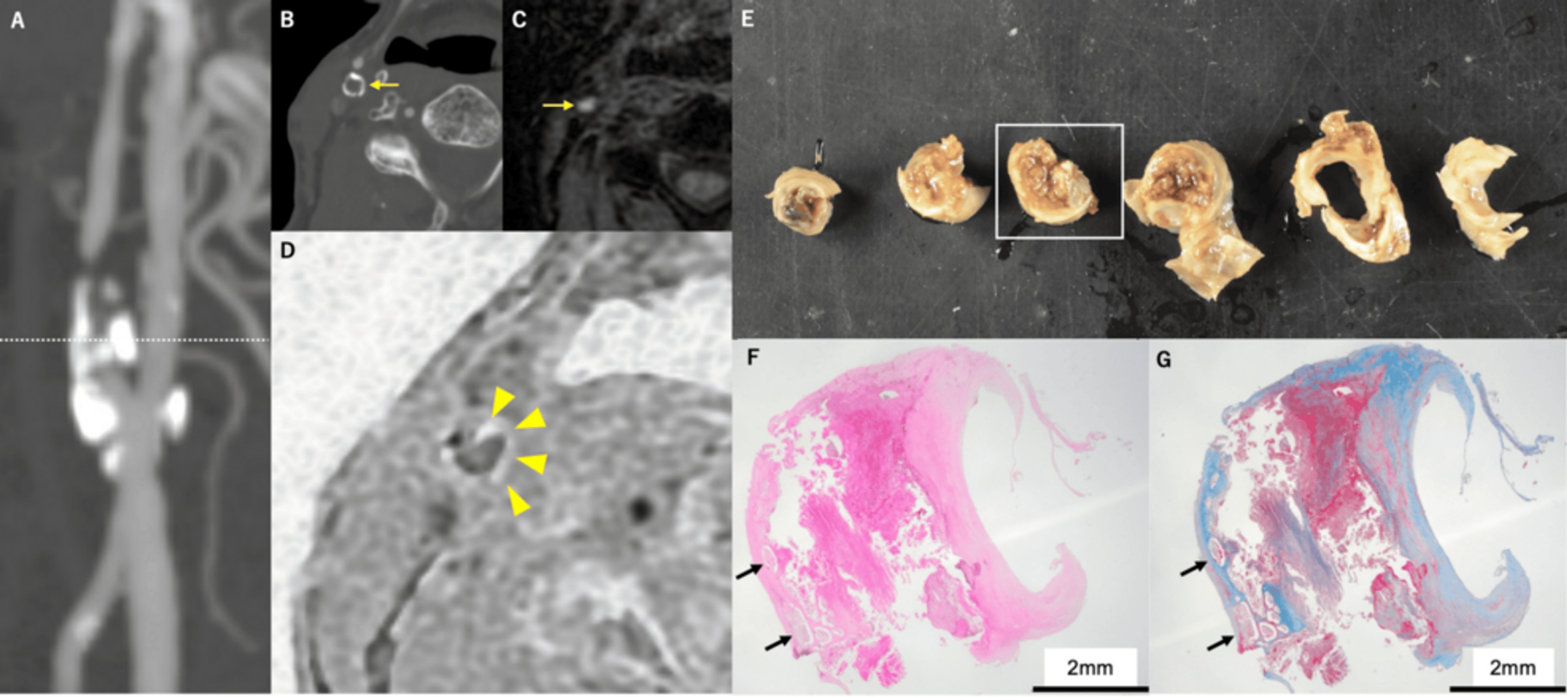
Representative case of the rim sign. A: CT angiography (CTA). B: CTA of the cross-sectional image of the stenotic part. Circumferential calcification of the outer membrane is visualized (yellow arrow). C: T1-weighted volume isotropic turbo spin echo acquisition (T1-CUBE) sequence for the cross-sectional image of the stenotic part. The unstable component is well visualized as a high-intensity lesion, but calcification is not clearly visualized (yellow arrow). D: Modified Liver Acquisition With Volume Acceleration (mLAVA) sequence for the cross-sectional image of the stenotic part. The carotid rim sign is well visualized as a high-intensity lesion (yellow arrowheads). E: Incised slices of the carotid endarterectomy specimen. F: Calcification is located along the outer border of the plaque (hematoxylin-eosin staining, arrowheads). G: Calcification is located within the outer fibrous layer of the plaque (Masson-trichrome staining, arrowheads). (F) and (G) are histological images corresponding to the open square area of (E).

A 54-year-old male with a medical history of hypertension and hyperlipidemia presented to our institution with amaurosis fugax. MRI showed right carotid artery stenosis. CTA revealed circumferential calcification of the outer membrane at the proximal internal carotid artery (ICA) (Figures 1A, 1B). High-resolution T1-weighted imaging (T1-CUBE) identified a high-signal area within the lesion, indicative of an unstable plaque; hence, we recognized it as a positive rim sign (Figure 1C). Additionally, the mLAVA sequence confirmed a high signal intensity at the same location (Figure 1D). Measurement of the stenosis using the North American Symptomatic Carotid Endarterectomy Trial (NASCET) criteria revealed a severe stenosis of 80% [8]. The patient subsequently underwent CEA. The plaque showed intraplaque hemorrhage, and pathological examination confirmed the presence of outer membrane calcification (Figures 1E-1G). Postoperative CTA showed complete resolution of the plaque. The patient’s postoperative course was uneventful, and he was discharged with a modified Rankin Scale (mRS) score of 0 at 10 days after CEA [9]. Four months after surgery, the course has been uneventful with no restenosis, and he continues to be followed up in the outpatient department.

Case 2: calcified nodule

**Figure 2:**
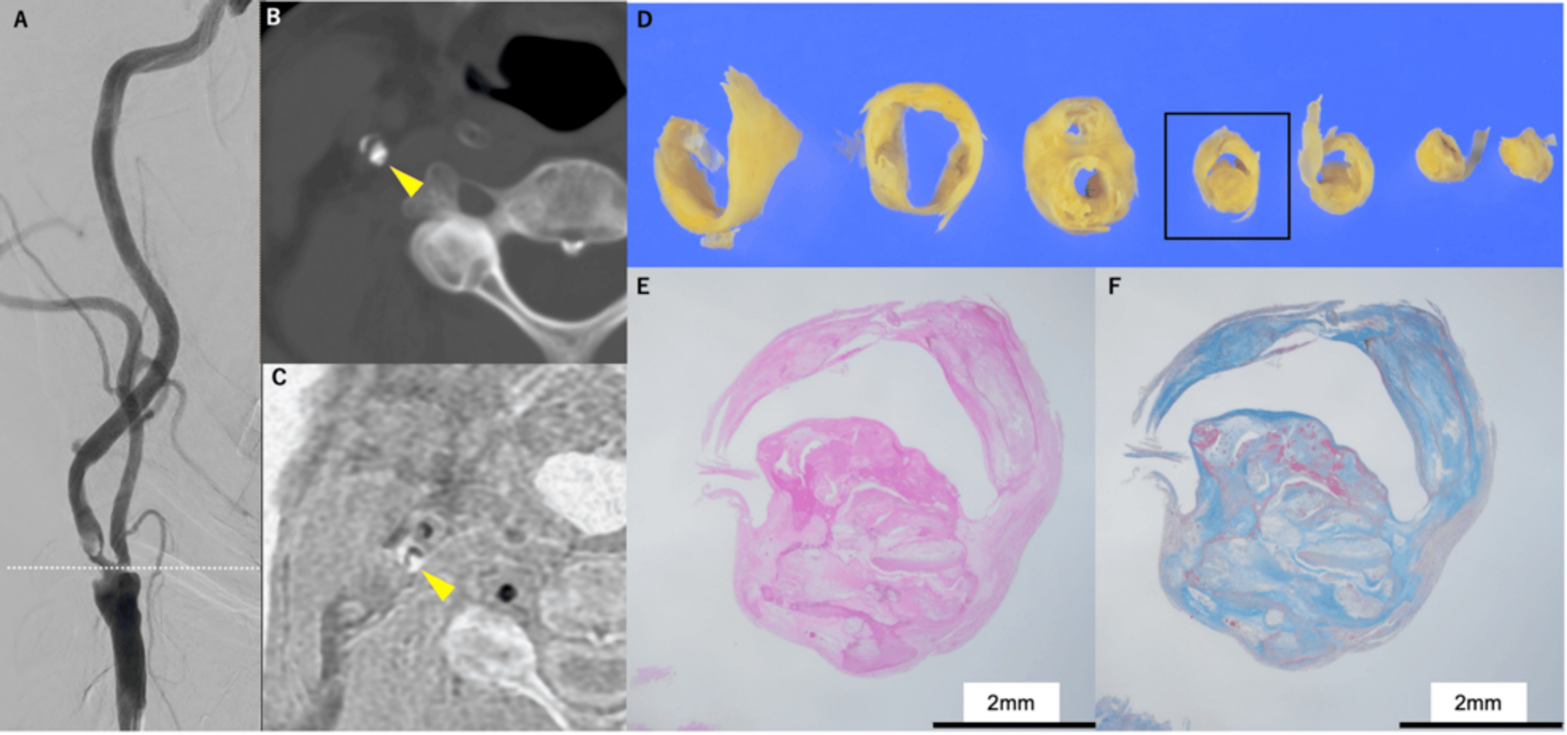
Representative case of a calcified nodule (CN). A: Digital subtraction angiography. B: Plain CT image for the cross-sectional image of the stenotic part. CN is visualized as a high-density area, but the relationship between CN and the lumen is unclear (yellow arrow). C: Modified Liver Acquisition With Volume Acceleration (mLAVA) sequence for the cross-sectional image of the stenotic part. CN is clearly protruded into the lumen as a high-intensity lesion (yellow arrow). D: Incised slices of the carotid endarterectomy specimen. E: The histopathological finding shows a calcified lesion with nodular calcification protruding into the lumen area (hematoxylin-eosin stain). F: Fibrin cap is present on the surface of the protruded CN  (Masson-trichrome staining) (E) and (F) are histological images corresponding to the open square area of (D).

An 82-year-old male with asymptomatic right ICA stenosis, which was progressively narrowing during the follow-up of carotid ultrasound, was evaluated for potential therapeutic intervention. His medical history included hypertension, hyperlipidemia, diabetes, and angina. Eight years ago, he had undergone coronary artery bypass grafting. Due to chronic kidney disease, CTA could not be performed, and plaque assessment was conducted by MRI. Plaque imaging technique of the mLAVA sequence revealed that three-quarters of the circumference of the vessel was a high-signal area at the origin of the ICA. Notably, a high signal region protruding into the vascular lumen was observed, suggesting a calcified nodule (Figures 2A-2C). Measurement of the stenosis using NASCET criteria revealed a severe stenosis of 71% [8]. CEA was chosen as the treatment for carotid artery stenosis. Intraoperative findings were protruding calcification, consistent with preoperative imaging. The pathological examination confirmed the presence of a CN (Figures 2D-2F). Postoperative CTA demonstrated complete resolution of the calcified plaque. The patient’s recovery was uneventful, and he was discharged on postoperative day 14 with an mRS score of 0 [9]. Five months after surgery, the course has been uneventful with no restenosis, and he continues to be followed up in the outpatient department.

## Discussion

We showed two types of calcified plaques, which may be a risk of ischemic events, using the mLAVA sequence. This is the first case report reporting this sequence. The characteristics of carotid plaques are important when assessing stroke risk factors. While calcified lesions have traditionally been considered stable plaques [1], calcification, such as the plaques described in this report, may cause plaque rupture and might be risk factors for stroke [10].

In Case 1, a positive rim sign was defined as adventitial calcification (<2 mm thick) with internal soft plaque (≥2 mm thick) [11]. It has been reported that patients with a positive rim sign have a higher prevalence of intraplaque hemorrhage and an increased risk of ischemic events [3,4]. Furthermore, we reported that the presence of a rim sign significantly elevates the risk of recurrent ischemic events [12]; hence, we suggest that patients with a positive rim sign require careful follow-up.

In case 2, a CN was pathologically defined as “a lesion with fibrous cap disruption and luminal thrombus associated with eruptive, dense, calcific nodules” [13]. Although CNs are the least common cause of acute coronary syndrome (ACS), they still account for 5% of all cases [14]. In a study, the prevalence of CNs was 2.8% among the specimens that were studied, and the proportion of women was significantly higher in the CN group (64.3%) [15].

Recently, with the development of intravascular imaging in coronary lesions such as optical coherence tomography, CNs have been classified into the following types: eruptive CNs are characterized by fibrous cap disruption with overlying thrombosis, and noneruptive CNs are characterized by a smooth intact fibrous cap without overlying thrombosis [8,16,17]. It has been reported that eruptive CNs cause ACS more often, and that they are more deformable and can get greater stent expansion but cause in-stent restenosis (ISR) more frequently than noneruptive CNs [16,17].

In the setting of carotid artery stenosis, there have been reported cases in which carotid artery stenting was unsuccessful due to inadequate stent expansion; subsequently, CEA was performed [18]. Although no studies have specifically evaluated the differences between eruptive and noneruptive CNs in carotid artery stenosis, we evaluated a noneruptive CN in Case 2 based on the coronary CN classification. This is because the pathological examination showed a smooth, intact fibrous cap without overlying thrombosis. Given this, it might be that carotid artery stenosis might have resulted in an unsuccessful outcome in Case 2.

Evaluation of calcified lesions has primarily been evaluated using CTA; however, exposure to ionizing radiation has been associated with an increased lifetime risk of cancer [5,19]. Additionally, the use of iodinated contrast media has the potential risk of renal dysfunction, which is particularly true for patients with carotid artery stenosis, who have a high prevalence of renal dysfunction [5,6]. The availability of MRI plaque imaging as an alternative can avoid these risks. Moreover, the mLAVA can be acquired within the same protocol as plaque imaging, designed to evaluate intraplaque hemorrhage. In the present study, the mLAVA sequence demonstrated morphological concordance with CTA in detecting calcified lesions, particularly with respect to lesion shape and anatomical location. CTA remains superior for absolute Hounsfield unit measurement. However, the sensitivity of mLAVA for detecting microcalcifications remains limited. Further advancements that enable precise visualization of fine calcified lesions would enhance the clinical utility of mLAVA.

## Conclusions

In this study, we presented the mLAVA sequence as a method for detecting calcified lesions using MRI, supported by histopathological correlation. Recently, there has been increased attention to the stroke risk associated with calcified lesions. We believe that MRI-based plaque imaging with the mLAVA could serve as an alternative option, particularly in patients who cannot undergo contrast-enhanced CT.
